# Impact of diverse aerobic exercise plans on glycemic control, lipid levels, and functional activity in stroke patients with type 2 diabetes mellitus

**DOI:** 10.3389/fendo.2024.1389538

**Published:** 2024-09-18

**Authors:** Kangcheng Chen, Yulong Wang, Dongxia Li, Jun Li, Yong Huang, Meiling Huang, Haifeng Ma

**Affiliations:** ^1^ School of Athletic Performance, Shanghai University of Sport, Shanghai, China; ^2^ Department of Rehabilitation, The First Affiliated Hospital of Shenzhen University, Shenzhen Second People’s Hospital, Shenzhen, China

**Keywords:** stroke, type 2 diabetes mellitus, aerobic exercise training, continuous glucose monitoring, glycemic variability, functional activity, lipid levels

## Abstract

**Aims:**

This study aimed to assess the effects of Low-to-Moderate Intensity Continuous Training (LMICT), Moderate-Intensity Interval Training (MIIT), and Reduced-Exertion High-Intensity Training (REHIT) on blood glucose regulation, functional recovery, and lipid levels in individuals who have experienced a stroke and are diagnosed with Type 2 Diabetes Mellitus (T2DM).

**Methods:**

Forty-two T2DM stroke patients were randomly allocated to four groups: LMICT, MIIT, REHIT, and a control group (CON). Participants continuously monitored their blood glucose levels throughout the intervention using continuous glucose monitoring (CGM) devices. The study comprised two exercise intervention cycles: the first lasting from Day 3 to Day 14 and the second from Day 15 to Day 28, with the initial two days serving as contrasting periods. Primary outcomes encompassed CGM-derived blood glucose measurements, the Barthel Index (BI), Fugl-Meyer Assessment lower-extremity subscale (FMA-LE), and alterations in triglycerides (TG), total cholesterol (TC), high-density lipoprotein cholesterol (HDL-c), and low-density lipoprotein cholesterol (LDL-c).

**Results:**

Compared with the CON, the MIIT group showed significant improvements in mean glucose (MG), glucose standard deviation (SD), time above range (TAR), and time in range (TIR). The REHIT group exhibited significantly reduced time below range (TBR), glucose SD, and coefficient of variation (CV). Regarding lipid levels, although the REHIT group achieved a significant reduction in TG levels compared with the CON, the overall effects of LMICT, MIIT, and REHIT on lipid profiles were relatively modest. Concerning functional recovery, the REHIT group significantly improved the BI and FMA-LE.

**Conclusion:**

Although the short-term quantitative impact of exercise on lipid levels may be limited, both REHIT and MIIT significantly improved glycemic management and reduced glucose variability in post-stroke patients with Type 2 Diabetes Mellitus. Additionally, REHIT notably enhanced functional recovery.

## 1 Introduction

Stroke is a leading cause of mortality among Chinese residents, accounting for one-third of global stroke-related fatalities and representing a significant challenge to China’s public health system (1, 2). Abnormal blood glucose levels have been identified as a significant risk factor for stroke, not only increasing disease complexity but also amplifying management challenges (3–5). In China, approximately 68.7% of stroke patients demonstrate glucose metabolism abnormalities, with 42.3% being concurrently diagnosed with diabetes (6). This proportion underscores the prevalence of diabetes among stroke patients and its potential impact on patient outcomes.

Glycated hemoglobin (HbA_1c_) has been the primary target for the clinical management of hyperglycemia in patients with type 2 diabetes mellitus (T2DM), reflecting mean glucose (MG) levels over the past two to three months. However, its correlation with stroke recurrence risk appears limited. The effectiveness of intensified blood glucose control (i.e., HbA_1c_ ≤ 7%) in preventing recurrent strokes remains unclear for patients with diabetes in the post-acute phase (7). This ambiguity likely arises from the close association between elevated MG levels and microvascular complications of diabetes, while the association with macrovascular complications seems more indirect (8). Additionally, HbA_1c_ cannot capture blood glucose fluctuations or provide information about glucose dynamics. However, advancements in continuous glucose monitoring (CGM) technology allow for a more precise assessment of an individual’s blood glucose status, offering real-time data on fluctuations, hyperglycemia, hypoglycemia, and overall glucose variability—crucial factors for diabetes treatment (9–11). Research demonstrates a clear association between time in range (TIR), time below range (TBR), and the risk of cardiovascular and cerebrovascular diseases in patients with diabetes (10, 12). Furthermore, prospective and retrospective cohort studies reveal a dose-response relationship between glycemic variability and stroke risk, emphasizing the practical significance of evaluating blood glucose levels in stroke patients using CGM-derived glycemic management indicators (13).

Effective blood glucose management is essential for the functional recovery of stroke patients, as it promotes neurovascular repair and functional improvement (14). Aerobic exercise has been established as a powerful secondary prevention strategy, reducing cardiovascular risks for post-stroke patients and managing complications associated with diabetes (15–18). The current guidelines from the American Heart Association/American Stroke Association recommend that stroke survivors engage in Low-to-Moderate Intensity Continuous Training (LMICT) three to five times per week, with each session lasting 20 to 60 minutes at an intensity of 40% to 70% of heart rate reserve, including five to ten minutes of lower-intensity warm-up and cool-down activities (19). Moreover, the American College of Sports Medicine and the American Diabetes Association recommend that patients with T2DM participate in at least 150 minutes of moderate-intensity or 75 minutes of high-intensity exercise weekly (20). However, physical disabilities frequently hinder stroke patients’ ability to adhere to these exercise recommendations. This situation can impede functional recovery and potentially increase the risk of stroke recurrence due to inadequate blood sugar control (18).

Although Reduced-Exertion High-Intensity Training (REHIT) and Moderate-Intensity Interval Training (MIIT) have shown promise in improving insulin sensitivity and reducing blood glucose levels in non-stroke populations, their effects on individuals with concurrent stroke and diabetes have not been extensively explored (21, 22). Therefore, this study aims to investigate the effects of LMICT, MIIT, and REHIT on CGM-derived indicators in stroke patients with diabetes while also tracking changes in essential prognostic indicators, such as lipid levels and functional activity. Additionally, the study examines the influence of different intensity levels and compares intermittent versus continuous exercise modalities in this high-risk population. The ultimate goal is to provide clinical evidence supporting the implementation of aerobic training programs for stroke patients with diabetes, facilitating improved glycemic control, lipid management, and functional recovery.

## 2 Materials and methods

### 2.1 Study participants

The trial enrolled 42 hospitalized patients concurrently diagnosed with stroke and T2DM. All participants provided written informed consent, and the study received approval from the regional Ethics Committee. The trial has been registered with the Chinese Clinical Trial Registry. Participant selection was based on the ‘2019 Diagnostic Essentials of Cerebrovascular Diseases in China’ for stroke (confirmed by imaging) and the ‘2020 Chinese Guidelines for Type 2 Diabetes’. Inclusion criteria included age 18-80 years, a stroke occurring between 15 days to 1 year prior, the ability to walk 10 meters independently (with or without assistance), and adequate cognitive function to engage in study activities. Exclusion criteria were: unstable vital signs, progressive or acute-phase stroke, transient ischemic attacks, history of brain injury or other central nervous system issues, major cardiac or pulmonary diseases, severe hepatic or renal dysfunction, musculoskeletal limitations, untreated deep venous thrombosis, severe diabetic complications, other serious concurrent illnesses, or involvement in other clinical trials.

### 2.2 Study design

**Figure 1:**
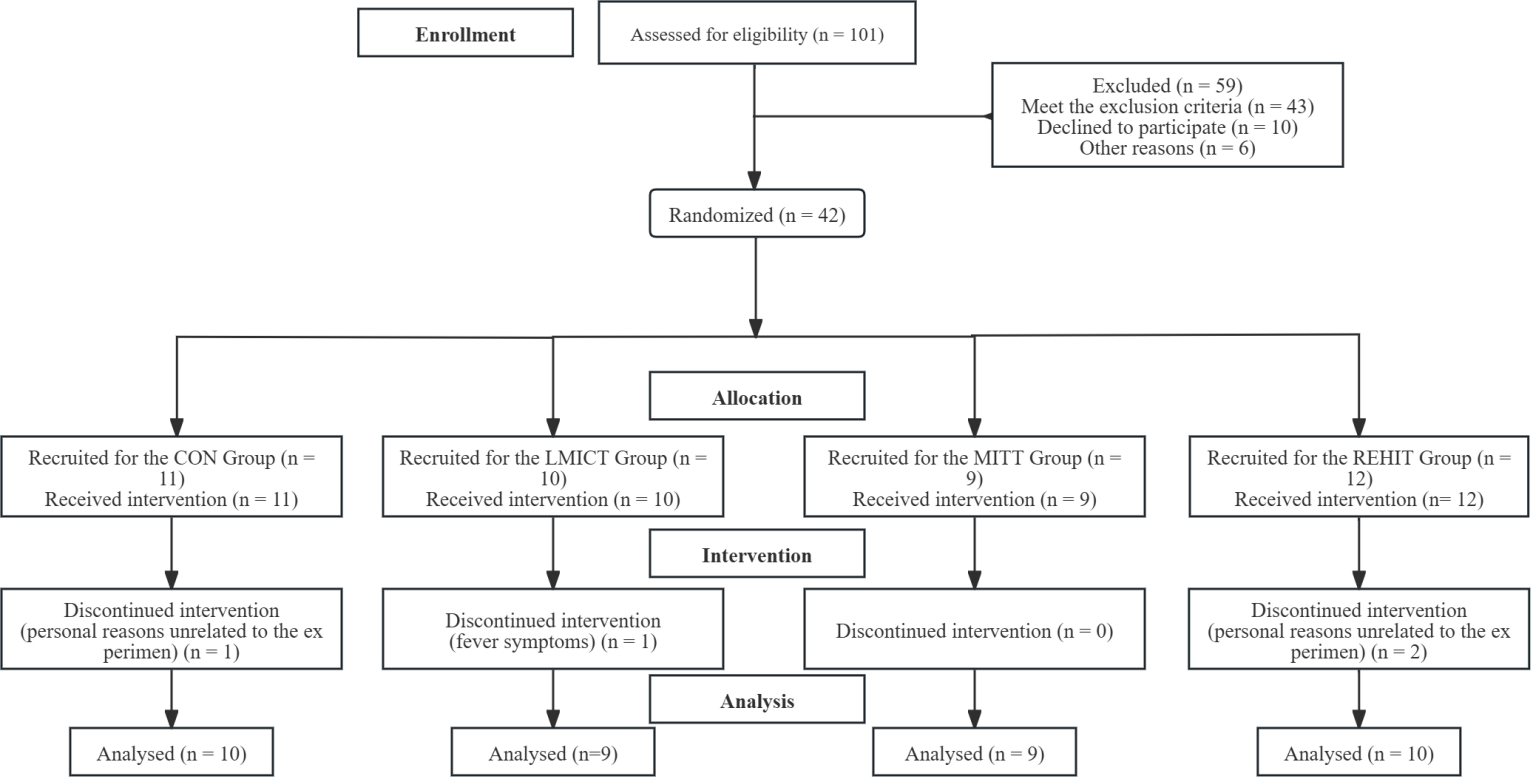
Flow diagram of the study participants.

**Figure 2:**
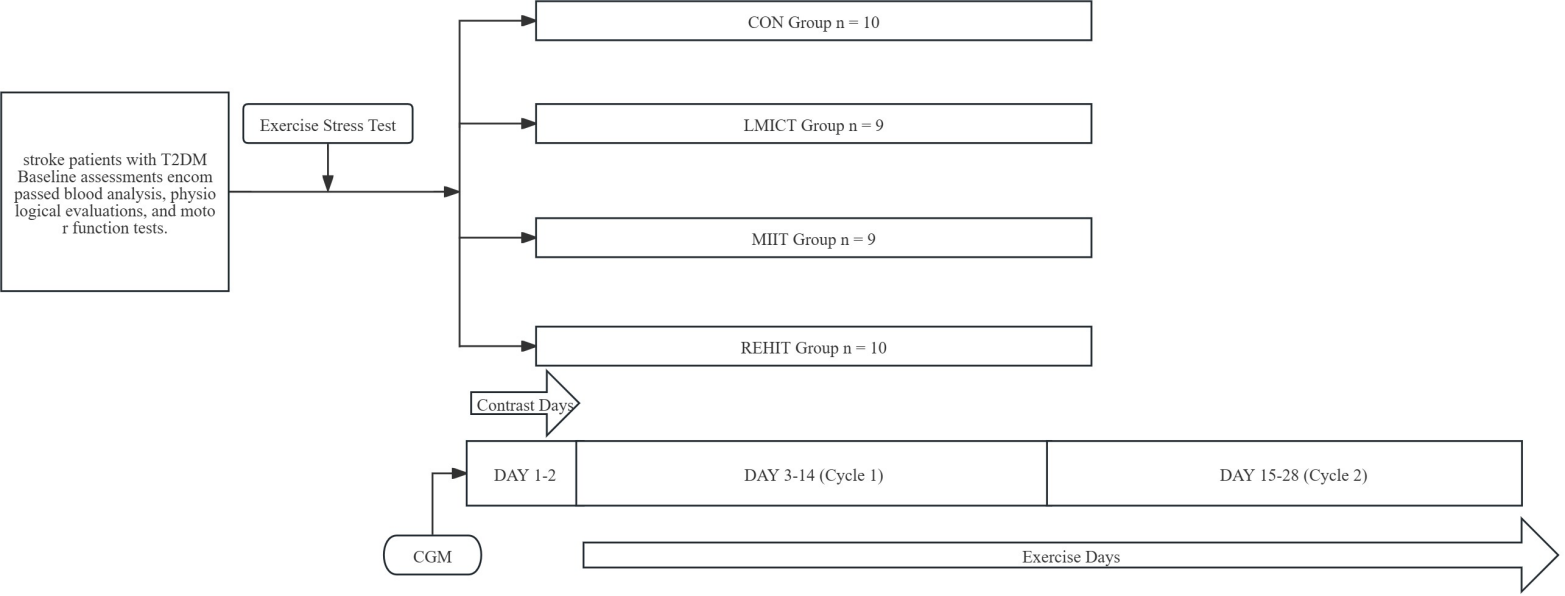
Study overview.

This prospective, randomized, controlled trial was conducted at the Rehabilitation Department of the Second People’s Hospital of Shenzhen in Guangdong Province between October 2022 and October 2023. At baseline, participants underwent a comprehensive assessment, including blood tests, physiological measurements, functional evaluations, and exercise stress testing. Following the baseline assessments, 42 participants were randomly allocated to four groups: three experimental groups and one control group. The participants then engaged in a 4-week intervention trial (**Figure 1**). The randomization process involved participants selecting and opening opaque sealed envelopes, each containing a specific intervention assignment. The first two days of the study were designated as the “Contrast Days,” and the data collected during this period were analyzed using mean values. “Cycle 1” referred to the period from day 3 to day 14, while “Cycle 2” encompassed the period from day 15 to day 28. Data analysis for these cycles was based on the mean values collected (**Figure 2**).

### 2.3 Sample size estimation

Metcalfe et al. reported that REHIT significantly reduced MG levels in patients with T2DM compared with a no-exercise control group, demonstrating a statistically significant Cohen’s *d* value of 0.55 (23). Their study employed a repeated measures design with three distinct phases: baseline Contrast Days (days 1-2), Cycle 1 (days 3-14), and Cycle 2 (days 15-28). In the current study, we transformed Cohen’s *d* to Cohen’s *f* using the approximate transformation *f* = *d*/2 (24), yielding a value of 0.275. To ensure a robust sample size while accounting for variability, we set the effect size at a relatively low-medium range (*f* = 0.25). The sample size was calculated using G*Power 3.1.9.7 software (Heinrich Heine Universität Düsseldorf, Düsseldorf, Germany), indicating that a total of 40 patients would be required to achieve 80% power at a significance level of 0.05. Given the small sample size, efforts will be made to minimize attrition. This study design enables the accurate assessment of the effects of LMICT, MIIT, and REHIT on glucose control in stroke patients with T2DM and the exploration of secondary outcomes related to functional recovery and lipid levels.

### 2.4 Collection of primary data

Critical clinical data collected included age, gender, height, weight, the duration of diabetes, the date of stroke onset, histories of smoking and alcohol consumption, medication use, and overall medical history. During baseline and endpoint assessments, participants provided fasting ante-cubital venous blood samples for the evaluation of fasting plasma glucose (FPG), glycated hemoglobin (HbA_1c_triglycerides (TG), total cholesterol (TC), high-density lipoprotein cholesterol (HDL-c), and low-density lipoprotein cholesterol (LDL-c). The glucose oxidase method was employed to quantitatively assess blood glucose concentration, while the scatter turbidity method was used to measure HbA_1c_ levels, with all samples analyzed in duplicate. Furthermore, the BI was utilized to evaluate patients’ activities of daily living, and the Fugl-Meyer Assessment lower-extremity subscale (FMA-LE) was administered to assess lower limb function.

### 2.5 Exercise stress test

In this study, participants underwent a symptom-limited maximum incremental cardiopulmonary exercise test using a four-limb coordinated rehabilitation training device (HNT-LE-E001A, Humaneutec, Guangzhou, China) and a cardiopulmonary exercise test system (Masterscreen CPX, ERGOLINE GMBH, Germany). Prior to testing, each participant was fitted with a 5-lead electrocardiogram (ECG), equipped with an automatic blood pressure monitoring cuff and a gas analysis mask, and the system was calibrated accordingly. Resistance was increased stepwise by 1-2 levels every 2 minutes based on the patient’s gender, age, height, weight, functional status, and exercise habits. The test protocol comprised several phases: 1)a 1-minute resting phase, during which the patient’s heart rate and blood pressure were recorded; 2) a 3-minute warm-up without resistance; 3) a load phase, during which resistance was gradually increased according to the pre-selected scheme, lasting for 8-12 minutes until either maximum exercise load was reached or termination criteria were met; 4) a recovery phase, consisting of 3 minutes of unloaded movement, followed by 3 minutes of rest, during which the recovery of blood pressure and heart rate was monitored. Each resistance level was maintained for 2 minutes throughout the exercise, with a constant pedaling speed of 80 steps/min (25). Termination criteria included: achieving a VO_2_ plateau (26); a heart rate exceeding 90% of the age-predicted maximum heart rate (or 85% for those on β-blockers) (27); a respiratory exchange ratio exceeding 1.0 (28); or a Borg scale rating (6–20) exceeding 17 (29). The test was also concluded if participants could not maintain the predetermined pedaling speed despite maximal effort.

### 2.6 Continuous glucose monitoring

In 2017, the International Conference on Advanced Technologies & Treatments for Diabetes (ATTD) established an international consensus stating that CGM should be conducted for a duration exceeding 14 days, with at least 10 days of valid data (30). Adhering to this consensus enables more effective monitoring of trends in participant blood glucose levels. Consequently, CGM occurred throughout the 28-day intervention period, and data were segmented into three stages for trend analysis: days 1-2, days 3-14, and days 15-28. On days 0 and day 14 of the experiment, participants had glucose sensors (GS1, SIBIONICS, Shenzhen, Guangdong, China) implanted in their non-dominant upper arm, each with a lifespan of 14 days. These sensors continuously recorded glucose levels every 5 minutes to obtain the MG, TIR, TBR, and TAR for each phase. Conventional analysis methods were employed to evaluate glycemic variability, constructing coefficients of variation (CV) and the standard deviation of glucose (SD glucose) based on the statistical characteristics of previous studies.

### 2.7 Medication therapy and dietary plan

All participants adhered to the endocrinologist’s recommendations for the administration of hypoglycemic medications. During the study, participants were directed to maintain their hypoglycemic treatment regimen without modifications, and the timing of hypoglycemic medications or insulin injections remained consistent on a daily basis. Regarding dietary intake, the caloric content and the proportions of carbohydrates, proteins, and fats in each meal were recorded for the two days preceding the experiment, referred to as the Contrast Days, based on a standardized meal plan. Under the guidance of therapists, participants were instructed to maintain the dietary patterns established during the Contrast Days for the following 22 days.

### 2.8 Exercise intervention program

**Figure 3:**
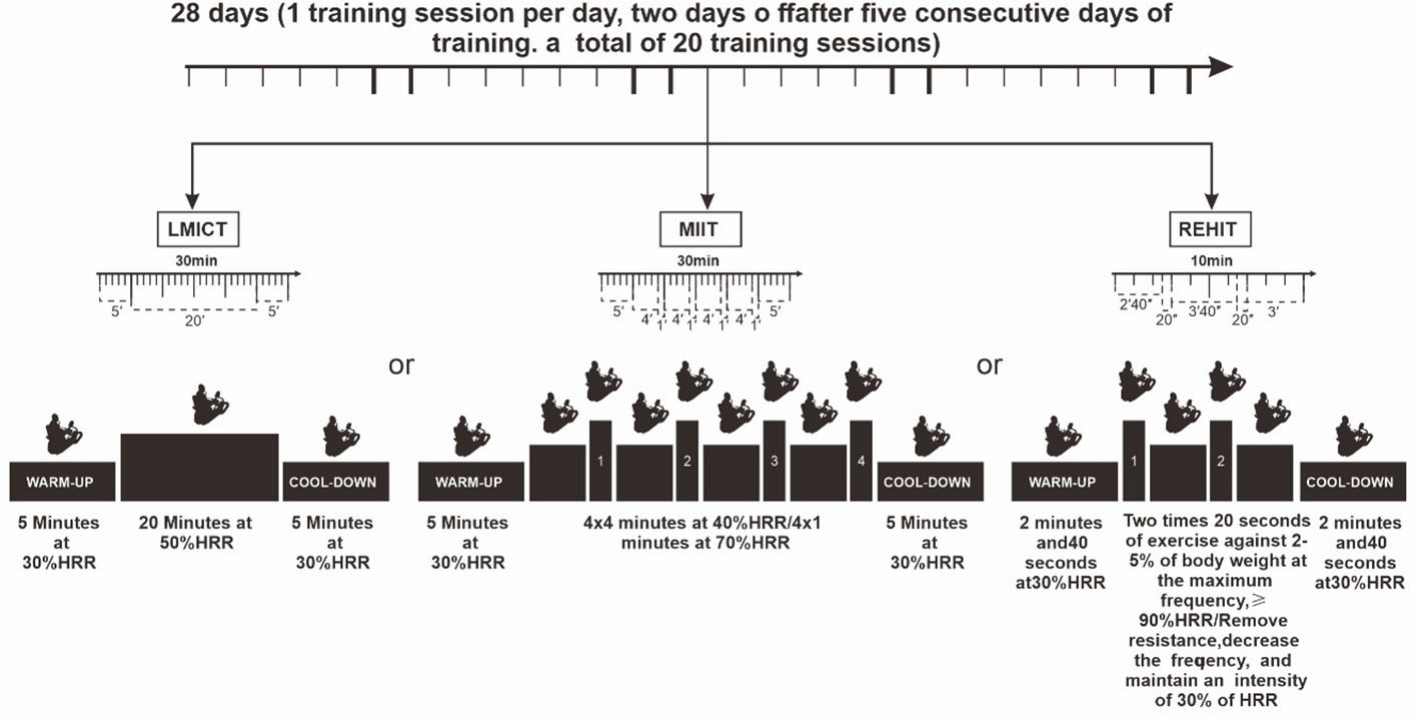
Exercise intervention program.

Under strict professional supervision, all exercise interventions were conducted to ensure their safety and efficacy. Participants in the experimental group used a standardized four-limb coordinated rehabilitation training device. Based on initial cardiopulmonary exercise test results, therapists customized the apparatus’s resistance levels and pedal speeds for each participant. Continuous heart rate monitoring was implemented throughout each session to maintain optimal exercise intensity, and participants rated their perceived exertion using the Borg scale (range 6-20) immediately after each session. The exercise intervention program started on day 3 and lasted until day 24, with daily sessions. The program included two rest days after every five consecutive exercise days to prevent overtraining, resulting in 20 sessions across the intervention period. The sessions were divided into two phases, each comprising 10 sessions. Participants in the control group (CON) continued their routine treatment regimen without participating in any structured exercise program (**Figure 3**).

### 2.9 Statistics

Data were analyzed using SPSS 23 software (IBM Corporation, Armonk, NY, USA). Categorical variables were presented as percentages, and continuous variables as mean ± standard deviation (M ± SD). Differences in baseline characteristics among the CON, LMICT, MIIT, and REHIT groups were evaluated using one-way analysis of variance (ANOVA) and the chi-square test. Levene’s test was applied to assess the homogeneity of variances, and if this assumption was violated, Welch’s ANOVA was used. Fisher’s exact test was employed for categorical variables when expected frequencies were less than five. Time and exercise mode were factors in a two-way repeated measures ANOVA for comparing and analyzing CGM data and deriving blood glucose indices for each exercise regimen. The Greenhouse-Geisser correction was applied when the assumption of sphericity was violated. Significant interaction effects were further analyzed using the Bonferroni method for pairwise comparisons. Additionally, the effects of the LMICT, MIIT, and REHIT exercise regimens on functional activity and lipid levels were assessed *via* analysis of covariance (ANCOVA), incorporating baseline TG, TC, HDL-c, LDL-c, BI, and FMA-LE as covariates, with Bonferroni correction for all pairwise comparisons. The significance level was set at *p* < 0.05.

## 3 Results

The study initially included 42 participants, distributed as follows: 11 in the CON group, 10 in the LMICT group, 9 in the MIIT group, and 12 in the REHIT group. Due to personal reasons unrelated to the experiment, 3 participants withdrew from the study, including one from the CON group and two from the REHIT group. Additionally, 1 participant from the LMICT group was excluded due to fever symptoms, rendering them unable to continue with the training. No participants reported adverse reactions during the LMICT, MIIT, and REHIT training sessions.

### 3.1 Participant characteristics

**Table 1** presents the participant characteristics. No statistically significant differences were observed among the LMICT, MIIT, REHIT, and CON groups in baseline characteristics, including age, height, weight, duration of diabetes, type of stroke, days post-stroke, glucose and lipid metabolism parameters, comorbid conditions, functional activity indicators, and types of concomitant treatments, such as oral hypoglycemic agents, insulin, statins, and β1-blockers.

**Table 1:** Demographic and baseline clinical characteristics.

	CON (n = 11)	LMICT (n = 10)	MIIT (n = 9)	REHIT (n = 12)	*P*
Withdraw halfway, n (%) Male/female, n (%) Age (years) Height (cm) Weight (kg) Duration of diabetes >10 years, n (%) HbA1c (%) FBG (mmol/L) Days since stroke onset Ischemic stroke, n (%) History of stroke, n (%) With hypertension, n (%) With dyslipidemia, n (%) With coronary artery disease, n (%) History of smoking, n (%) History of alcohol consumption, n (%) TG (mmol/L) TC (mmol/L) HDL-c (mmol/L) LDL-c (mmol/L) BI FMA-LE	1 (9.1%) 3 (27.3%) 59.73 ± 12.19 166.82 ± 5.88 63.27 ± 8.64 5 (45.5%) 8.11 ± 1.57 6.46 ± 1.48 65.27 ± 65.41 10 (90.9%) 2 (18.2%) 10 (90.9%) 1 (9.1%) 1 (9.1%) 4 (36.4%) 2 (18.2%) 1.27 ± 0.36 3.27 ± 1.24 0.97 ± 0.45 1.84 ± 0.61 58.18 ± 33.26 17.91 ± 7.78	1 (10%) 1 (10.0%) 62.20 ± 8.01 166.70 ± 4.37 61.70 ± 9.88 4 (40.0%) 7.52 ± 1.17 6.07 ± 1.14 100.00 ± 76.93 7 (70.0%) 0 (0.0%) 9 (90.0%) 1 (10.0%) 2 (20.0%) 6 (60.0%) 5 (50.0%) 1.54 ± 0.92 3.27 ± 0.91 1.00 ± 0.23 1.91 ± 0.58 53.00 ± 17.19 17.60 ± 7.85	0 (0%) 4 (44.4%) 63.67 ± 10.28 162.56 ± 8.65 58.33 ± 9.64 6 (66.7%) 8.35 ± 1.57 7.23 ± 1.97 102.3 ± 101.86 8 (88.9%) 2 (22.2%) 9 (100%) 1 (11.1%) 3 (33.3%) 3 (33.3%) 1 (11.1%) 1.38 ± 0.57 3.95 ± 1.14 1.07 ± 0.30 2.49 ± 1.00 55.00 ± 22.91 17.67 ± 7.07	2 (16.7%) 3 (25.0%) 57.42 ± 10.47 165.08 ± 8.12 63.75 ± 9.18 5 (41.7%) 7.70 ± 1.33 6.30 ± 1.16 121.67 ± 90.98 9 (75.0%) 2 (16.7%) 12 (100.0%) 2 (16.7%) 2 (16.7%) 4 (33.3%) 6 (50.0%) 1.10 ± 0.30 3.53 ± 0.89 1.16 ± 0.22 1.99 ± 0.60 57.92 ± 22.00 18.83 ± 8.04	0.894 0.426 0.536 0.598 0.571 0.675 0.550 0.523 0.461 0.580 0.561 0.707 1.000 0.603 0.598 0.126 0.323 0.453 0.491 0.189 0.938 0.980
Oral blood-glucose-lowering medication					
Biguanides, n (%) DPP-4 Inhibitors, n (%) SGLT2 Inhibitors, n (%) Sulfonylureas, n (%)	5 (45.5%) 2 (18.2%) 2 (18.2%) 3 (27.3%)	5 (50.0%) 3 (30%) 2 (20.0%) 4 (40.0%)	4 (44.4%) 3 (33.3%) 3 (33.3%) 3 (33.3%)	8 (66.7%) 3 (25.0%) 2 (16.7%) 5 (41.7%)	0.730 0.900 0.848 0.919
Insulin treatment n (%)					
Basal Insulin, n (%) Multiple Daily Injections, n (%) Biphasic Insulin, n (%) Statins, n (%) β1-blocker, n (%)	3 (27.3%) 3 (27.3%) 1 (9.1%) 10 (90.9%) 2 (18.2%)	2 (20.0%) 2 (28.6%) 1 (10.0%) 6 (60.0%) 4 (40.0%)	3 (33.3%) 3 (33.3%) 0 (0.0%) 6 (66.7%) 2 (22.2%)	3 (25.0%) 2 (16.7%) 1 (8.3%) 8 (66.7%) 4 (33.3%)	0.963 0.831 1.000 0.402 0.722

Nominal data are presented as n (%). There were no between-group differences at baseline.

### 3.2 analysis of continuous glucose monitoring data on contrast days in cycle 1 and cycle 2

**Figure 4:**
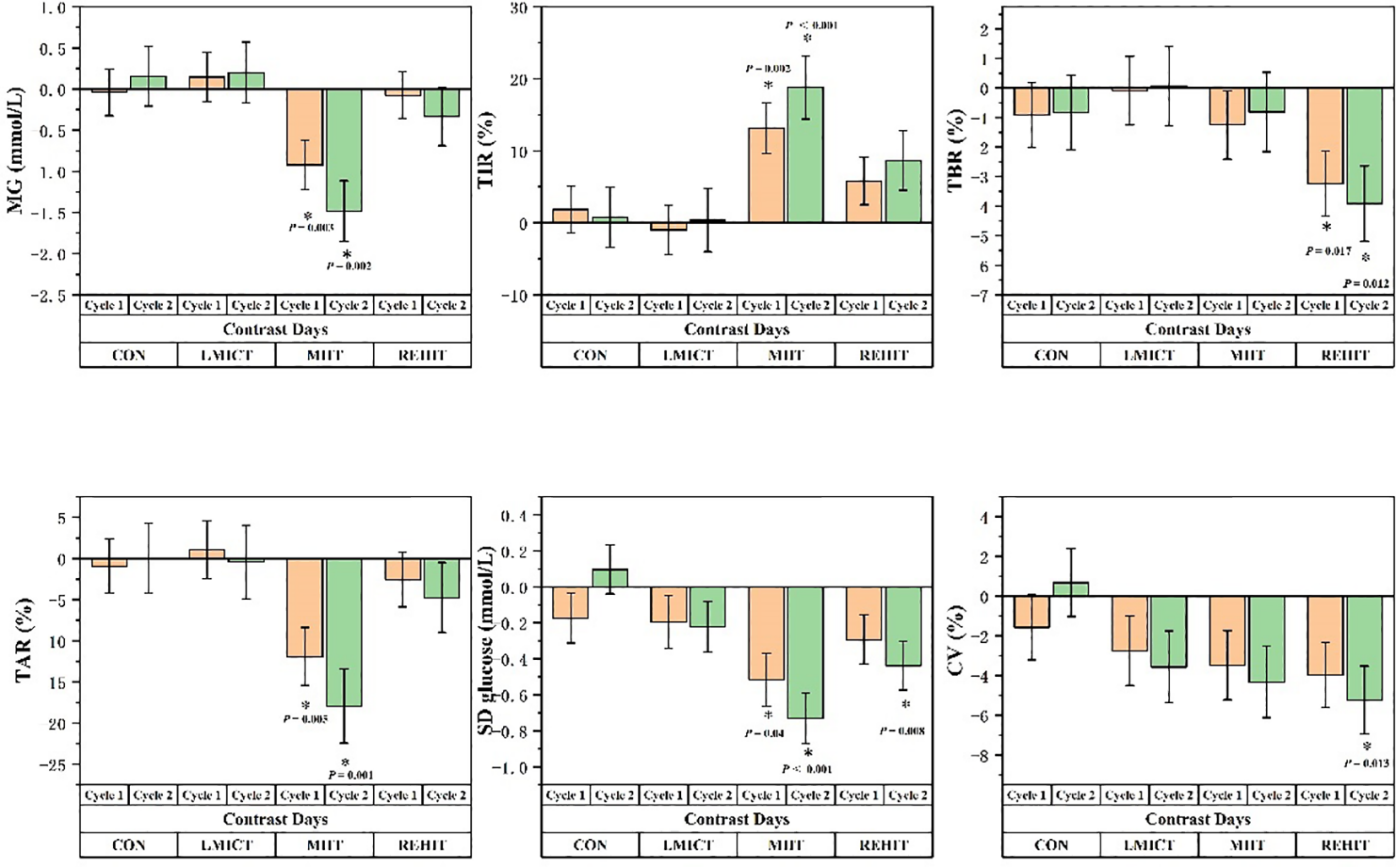
Panels display the variations in the least squares means of CGM-derived measurements relative to Contrast Days for each of Cycles 1 and 2 across the CON, LMICT, MIIT, and REHIT groups. The vertical axis depicts the difference in average values, and ‘I’ represents the standard error, with *p ≤ 0.05 indicating statistical significance.

The analysis of MG values indicated a significant main effect among groups (*F =* 3.110, *p* = 0.039), while the main effect of time was not statistically significant (*F =* 3.327, *p* = 0.065). However, a significant interaction between group and time was observed (*F* = 3.769, *p* = 0.011). Specifically, in the MIIT group, MG values showed a significant reduction over time (*F* = 8.343, *p* = 0.001). Multiple comparisons demonstrated that MG values significantly decreased from Contrast Days to Cycle 1 and Cycle 2. Relative to the Contrast Days, significant differences were observed between Cycle 1 [mean difference (MD) = -0.923, *p* = 0.013] and Cycle 2 (MD = -1.484, *p* = 0.001) (**Table 2**, **Figure 4**).

**Table 2:** Comparison of CGM-derived blood glucose monitoring data among participants on contrast days, cycle 1, and cycle 2.

		MG (mmol/L)	CV (%)	SD glucose (mmol/L)	TIR (%)	TBR (%)	TAR (%)
A: CON B: LMICT C: MIIT D: REHIT	Contrast Days Cycle 1 Cycle 2 Contrast Days Cycle 1 Cycle 2 Contrast Days Cycle 1 Cycle 2 Contrast Days Cycle 1 Cycle 2	8.45 ± 1.44 8.42 ± 1.55 8.61 ± 1.52 7.55 ± 0.95 7.70 ± 0.72 7.75 ± 0.73 8.45 ± 2.32 7.53 ± 1.01 6.96 ± 0.64 7.11 ± 1.26 7.03 ± 0.76 6.77 ± 0.56	27.32 ± 9.92 25.74 ± 7.04 28.00 ± 6.21 30.56 ± 6.47 27.80 ± 5.60 26.98 ± 6.46 29.93 ± 9.66 26.44 ± 5.57 25.61 ± 5.52 31.80 ± 6.40 27.83 ± 5.95 26.56 ± 4.46	2.34 ± 0.96 2.17 ± 0.68 2.44 ± 0.76 2.34 ± 0.66 2.14 ± 0.50 2.11 ± 0.61 2.53 ± 0.97 2.01 ± 0.55 1.80 ± 0.46 2.24 ± 0.45 1.95 ± 0.43 1.80 ± 0.37	72.61 ± 20.12 74.46 ± 19.03 73.37 ± 21.09 83.21 ± 10.71 82.22 ± 8.76 83.57 ± 9.10 70.51 ± 26.03 83.66 ± 10.41 89.28 ± 6.48 82.39 ± 11.41 88.20 ± 8.44 91.04 ± 5.88	1.86 ± 3.96 0.94 ± 1.08 1.03 ± 1.52 1.10 ± 1.48 1.01 ± 0.96 1.17 ± 1.20 3.19 ± 3.53 1.94 ± 2.00 2.38 ± 1.90 5.58 ± 8.70 2.34 ± 4.31 1.66 ± 2.23	25.54 ± 19.52 24.61 ± 19.18 25.60 ± 21.02 15.69 ± 9.83 16.77 ± 8.57 15.26 ± 8.39 26.31 ± 27.18 14.40 ± 10.14 8.35 ± 6.10 12.04 ± 10.38 9.46 ± 7.17 7.29 ± 4.59
Between-group comparison Within-subject comparison Interaction effect	*F*, *P* *F*, *P* *F*, *P*	3.110, 0.039 3.327, 0.065 3.769, 0.011	0.174, 0.913 10.514, < 0.001 1.577, 0.187	0.485, 0.695 16.737, < 0.001 4.199, 0.004	1.851, 0.157 9.297, 0.002 3.372, 0.016	1.083, 0.370 4.656, 0.030 1.598, 0.197	2.421, 0.083 5.674, 0.013 3.024, 0.026

Regarding TIR values, while the main effect among groups was not significant (*F* = 1.851, *p* = 0.157), a significant main effect of time was detected (*F* = 9.297, *p* = 0.002), along with a significant interaction effect observed between time and group (*F* = 3.372, *p* = 0.016). In the MIIT group, TIR values increased significantly over time (*F* = 8.947, *p* = 0.001). Multiple comparisons indicated significant increases in TIR values from Contrast Days to Cycle 1 (MD = 13.151, *p* = 0.002) and Cycle 2 (MD = 18.771, *p* < 0.001) (**Table 2**, **Figure 4**).

The analysis of TBR values indicated that the main effect among groups was not significant (*F* = 1.083, *p* = 0.370), but the main effect of time proved significant (*F* = 4.656, *p* = 0.030), with no significant interaction between the group and time (*F* = 1.598, *p* = 0.197). In the REHIT group, TBR values significantly decreased over time (*F* = 4.625, *p* = 0.017). Multiple comparisons revealed significant reductions in TBR values from Contrast Days to Cycle 1 (MD = -3.238, *p* = 0.017) and Cycle 2 (MD = -3.912, *p* = 0.012) (**Table 2**, **Figure 4**).

The analysis of TAR values indicated that the main effect among groups was not significant (*F* = 2.421, *p* = 0.083), but the main effect of time proved significant (*F* = 5.674, *p* = 0.013), alongside a significant interaction effect observed between time and group (*F* = 3.024, *p* = 0.026). In the MIIT group, TAR values showed a significant decline over time (*F* = 7.803, *p* = 0.002). Multiple comparisons showed significant decreases in TAR values from Contrast Days to Cycle 1 (MD = -11.902, *p* = 0.005) and Cycle 2 (MD = -17.958, *p* = 0.001) (**Table 2**, **Figure 4**).

The analysis of the SD glucose indicated no significant main effect among groups (*F* = 0.485, *p* = 0.695), but the main effect of time was significant (*F* = 16.737, *p* < 0.001), with a significant interaction effect observed between time and group (*F* = 4.199, *p* = 0.004). Specifically, significant reductions in SD glucose over time were observed in the CON, MIIT, and REHIT groups (*F* = 5.970, *p* = 0.006; *F* = 13.884, *p* < 0.001; and *F* = 5.789, *p* = 0.007, respectively). Moreover, multiple comparisons revealed that in the CON condition, SD glucose significantly increased from Cycle 1 to Cycle 2 (MD = 0.271, *p* = 0.004), while in the MIIT group, SD glucose significantly decreased from Contrast Days to Cycle 1 (MD = -0.516, *p* = 0.004) and Cycle 2 (MD = -0.731, *p* < 0.001). In the REHIT group, SD glucose significantly decreased from Contrast Days to Cycle 2 (MD = -0.439, *p* = 0.008) (**Table 2**, **Figure 4**).

Finally, the analysis of the CV values revealed no significant main effect among groups (*F* = 0.174, *p* = 0.913). However, the main effect of time proved significant (*F* = 10.514, *p* < 0.001), with no significant interaction effect observed between group and time (*F* = 1.577, *p* = 0.187). In the REHIT group, CV values significantly decreased over time (*F* = 4.562, *p* = 0.018). Multiple comparisons indicated that in the REHIT group, CV values significantly decreased from Contrast Days to Cycle 2 (MD = -5.235, *p* = 0.013) (**Table 2**, **Figure 4**).

### 3.3 Pre- and post-study analysis of blood lipid and functional test variations

**Figure 5:**
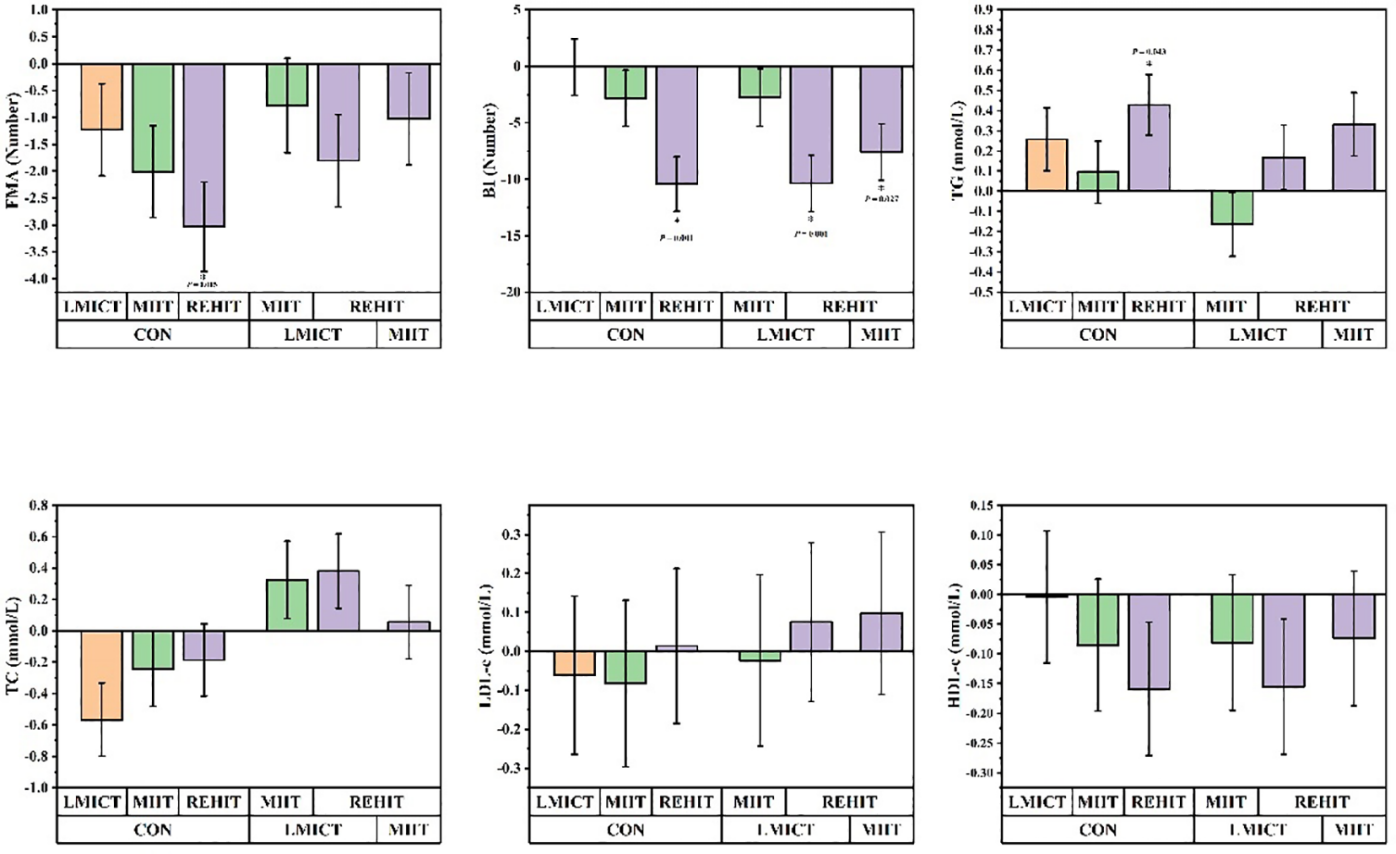
Panels display the least squares mean changes pre- and post-study for the CON group compared with the LMICT, MIIT, and REHIT groups. ‘I’ denotes the standard error, with *p ≤ 0.05 denoting statistical significance.

Functional outcomes, as assessed by the BI, showed mean improvements of 6.11, 8.87, 16.46, and 6.05 points in the LMICT, MIIT, REHIT, and CON groups, respectively. *Post-hoc* analyses revealed a significant improvement in the REHIT group compared with the CON group, with a least squares MD of 10.41 points (95% CI: 3.62 to 17.21, *p* = 0.001). Concurrently, FMA-LE demonstrated mean improvements of 2.43, 3.21, 4.23, and 1.20 points in the LMICT, MIIT, REHIT, and CON groups, respectively. The REHIT group exhibited a significant improvement over CON, with a least squares MD of 3.03 points (95% CI: 0.70 to 5.37, *p* = 0.005) (**Table 3**, **Figure 5**).

**Table 3:** Comparison of blood lipid and functional test indicators among participants before and after the start of the study indicators.

	CON	LMICTLMICT	MITT	REHIT
BI (number) Least-squares mean change ± SE between exercise day and Contrast Days Least-squares mean difference as compared with CON (95% CI) FMA-LE (number) Least-squares mean change ± SE between exercise day and Contrast Days Least-squares mean difference as compared with CON (95% CI) TG (mmol/L) Least-squares mean change ± SE between exercise day and Contrast Days Least-squares mean difference as compared with CON (95% CI) TC (mmol/L) Least-squares mean change ± SE between exercise day and Contrast Days Least-squares mean difference as compared with CON (95% CI) HDL-c (mmol/L) Least-squares mean change ± SE between exercise day and contrast day Least-squares mean difference as compared with CON (95% CI) LDL-c (mmol/L) Least-squares mean change ± SE between exercise day and Contrast Days Least-squares mean difference as compared with CON (95% CI)	6.05 ± 1.71 1.20 ± 0.59 0.13 ± 0.11 -0.25 ± 0.16 -0.01 ± 0.08 -0.09 ± 0.14	6.11 ± 1.81 0.06 (-6.93; 7.05) 2.43 ± 0.62 1.23 (-1.17; 3.63) -0.13 ± 0.11 -0.26 (-0.70; 0.18) 0.31 ± 0.17 0.57 (-0.09; 1.22) -0.01 ± 0.08 0.00 (-0.31; 0.31) -0.03 ± 0.15 0.06 (-0.51; 0.63)	8.87 ± 1.80 2.82 (-4.16; 9.80) 3.21 ± 0.62 2.01 (-0.39; 4.41) 0.03 ± 0.11 -0.10 (-0.53; 0.34) -0.01 ± 0.17 0.24 (-0.43; 0.92) 0.08 ± 0.08 0.09 (-0.23; 0.40) -0.00 ± 0.16 0.08 (-0.52; 0.68)	16.46 ± 1.72 10.41 (3.62; 17.21) 4.23 ± 0.59 3.03 (0.70; 5.37) -0.30 ± 0.11 -0.43 (-0.85; -0.01) -0.07 ± 0.16 0.19 (-0.46; 0.83) 0.15 ± 0.08 0.16 (-0.16; 0.47) -0.10 ± 0.14 -0.01 (-0.57; 0.54)

Throughout the study, the least squares mean of TG exhibited the following changes: it decreased by 0.13 mmol/L in the LMICT group, increased by 0.03 mmol/L in the MIIT group, decreased by 0.30 mmol/L in the REHIT group, and increased by 0.13 mmol/L in the CON group, pre- and post-study. *Post-hoc* analyses revealed that, compared to CON, the REHIT group exhibited a significant decrease in TG levels of 0.43 mmol/L (95% CI: -0.85 to -0.01, p = 0.043). The least squares mean of TC demonstrated the following changes: it increased by 0.31 mmol/L in the LMICT group, decreased by 0.01 mmol/L in the MIIT group, decreased by 0.07 mmol/L in the REHIT group, and decreased by 0.25 mmol/L in the CON group. *Post-hoc* analyses determined that changes in TC among the intervention groups, relative to the CON group, were not statistically significant. By the end of the study, the least squares mean of HDL-c shifted as follows: it declined slightly by 0.01 mmol/L in the LMICT group, increased by 0.08 mmol/L in the MIIT group, increased by 0.15 mmol/L in the REHIT group, and decreased by 0.01 mmol/L in the CON group. *Post-hoc* analyses showed no statistically significant differences in HDL-c changes between the intervention groups and the CON group. The least squares mean of LDL-c changed as follows: it decreased by 0.03 mmol/L in the LMICT group, remained stable at approximately 0.00 mmol/L in the MIIT group, decreased by 0.10 mmol/L in the REHIT group, and decreased by 0.09 mmol/L in the CON group. *Post-hoc* analyses showed no statistically significant differences in LDL-c changes between the intervention groups and CON (**Table 3**, **Figure 4**).

## 4 Discussion

### 4.1 Assessing the tolerance and safety risks of LMICT, MIIT, and REHIT in stroke patients with diabetes

This study was conducted under professional supervision, utilizing a four-limb coordinated rehabilitation training device to effectively engage significant muscle groups in accordance with guideline recommendations (31). Supervision records demonstrated an absence of adverse reactions or negative emotions during the experimental process, suggesting good tolerability of the intervention measures. Notably, inappropriate exercise can result in adverse reactions, with hypoglycemic events being the most common. Hypoglycemia may exacerbate brain damage in stroke patients, potentially leading to more severe cerebral ischemic injury, cerebral edema, and, in extreme cases, irreversible brain damage. However, this study found that the interaction effect between time and group was not significant for the LMICT, MIIT, and REHIT groups (*F* = 1.598, *p* = 0.197). No significant differences in the increase of TBR were observed between the Contrast Days, Cycle 1, and Cycle 2. Additionally, TBR values in the REHIT group significantly decreased from the Contrast Days to Cycles 1 and 2 (MD = - 3.238, *p* = 0.017; MD = -3.912, *p* = 0.012). Although research indicates that patients with T2DM treated with insulin or insulin secretagogues (such as sulfonylureas and glinides) face a higher risk of hypoglycemia after prolonged high-intensity exercise (32–36), 30 to 40 minutes of moderate-intensity exercise do not significantly raise the risk of hypoglycemia, even in those on long-term insulin therapy (37). Moreover, short-term high-intensity exercise may also help prevent hypoglycemia (20). This study corroborates previous research, suggesting that daily 30-minute sessions of LMICT, MIIT, and 10-minute sessions of REHIT do not elevate the risk of hypoglycemia in patients with concurrent diabetes and stroke.

### 4.2 Impact of LMICT, MIIT, and REHIT on MG levels in stroke patients with diabetes

The prevailing view holds that MG levels are the primary factor leading to the glycation of hemoglobin and other proteins, which is considered the initial stage in developing long-term complications (38, 39). This study’s findings reveal a significant reduction in participants’ MG levels over time under MIIT conditions. Repeated measures ANOVA demonstrated the statistical significance of MG level improvements and the critical interaction between time and exercise type, emphasizing the influence of exercise patterns and accumulated exercise days on MG levels. This finding aligns with previous research assessing the effects of 40 minutes of low-intensity continuous exercise (60% of VO_2_ peak) and interval exercise (alternating 4 minutes at 50% of VO_2_ peak with 1 minute at 80% of VO_2_ peak) on glycated hemoglobin (HbA_1c_) levels, noting a significant decrease in the interval exercise group. Moreover, studies have shown that long-term exercise may gradually reduce weekly HbA_1c_ levels by 0.009% to 0.043% (22, 40). Despite the close correlation between HbA_1c_ and MG levels measured by CGM, research has revealed significant variances in MG levels among individuals with identical HbA_1c_ levels, potentially due to differences in individual hemoglobin glycation rates and other physiological factors (9, 41). Consequently, MG levels have emerged as a more sensitive metric than HbA_1c_ for evaluating the effects of short-term interventions. The results of this study suggest that MIIT, which combines moderate to high intensity and duration, offers significant advantages over other exercise regimens in improving short-term average blood glucose levels in patients with diabetes and concurrent stroke.

### 4.3 Impact of LMICT, MIIT, and REHIT on glycemic variability in stroke patients with diabetes

At the ATTD conference, TIR was recognized as the primary metric in CGM research. Extensive research has confirmed an association between lower TIR and increased risk factors for major vascular diseases (17). A 2021 longitudinal study strongly supported TIR as a protective factor against cardiovascular disease mortality (12). Meta-analyses have indicated that exercise therapy significantly improves TIR in patients with T2DM, with a weighted mean difference (WMD) of 4.21% (95% CI: 0.95 to 7.46%; *P* < 0.01) (42). Our study further elucidates the variability in the effectiveness of different exercise regimens in enhancing TIR. Our findings indicate that, although inter-group differences in TIR were insignificant, the main effect of time was highly significant.

Furthermore, the time and exercise group interaction displayed statistical significance, with MIIT exhibiting a distinct advantage in improving TIR. Importantly, even small TIR increases of 5% have substantial clinical implications and can considerably enhance glycemic control (30). With the exception of the LMICT group, the MIIT and REHIT groups demonstrated TIR improvements, surpassing 5% from Contrast Days to Cycle 2. Correspondingly, only the MIIT group showed a statistically significant reduction in TAR from Contrast Days to Cycle 2, potentially due to the strong inverse correlation between TIR and TAR (43). The study findings indicate that, while REHIT may have clinical significance in improving TIR among stroke patients with concurrent diabetes, MIIT is superior in decreasing hyperglycemia risk and increasing the duration patients maintain blood glucose levels within the target range. In contrast, LMICT appears ineffective in improving TIR or reducing TAR in this patient population.

### 4.4 Impact of LMICT, MIIT, and REHIT on glycemic variability in stroke patients with diabetes

Glycemic variability, commonly assessed using SD glucose, CV, or a combination thereof, is associated with an increased risk of recurrent cardiovascular events and mortality in patients with ischemic stroke and diabetes. Furthermore, glycemic variability is linked to brain atrophy and cognitive impairment in patients with T2DM, potentially exacerbating cognitive decline in stroke survivors (44, 45). SD glucose is a parameter reflecting daily blood glucose fluctuations, quantifying deviations from the mean level, and is a crucial indicator for assessing glycemic variability. Compared to Contrast Days, this study demonstrated a significant reduction in SD glucose for the MIIT and REHIT groups, indicating that physiological adaptations from sustained high-level exercise enhance blood glucose control. This enhancement is likely achieved by extending the duration within the target blood glucose range and minimizing the time spent at extreme glucose peaks. Compared to SD glucose, CV (calculated as SD glucose ÷MG) more accurately reflects deviations associated with hypoglycemia and significantly correlates with the TBR metric (9, 46). Although the specific mechanisms by which REHIT improves CV are not fully understood, previous research has demonstrated that 10 minutes of REHIT can reduce muscle glycogen by approximately 20% and increase the expression of peroxisome proliferator-activated receptor gamma coactivator 1-alpha (PGC-1α) and glucose transporter type 4 (GLUT4) mRNA in skeletal muscle. The rapid increase in PGC-1α is associated with enhanced mitochondrial biogenesis, elevated GLUT4 expression, and increased muscle glucose transport capacity. These exercise-induced adaptive changes indicate that the upregulation of PGC-1α and GLUT4 contributes to improved glucose uptake and could enhance glucose utilization efficiency by promoting fatty acid oxidation and enhanced muscle insulin sensitivity, thereby benefiting the control or reduction of hypoglycemia risk and maintaining stable blood glucose levels (47, 48). These findings highlight the beneficial effects of MIIT and REHIT on reducing glycemic variability in patients with stroke and concurrent T2DM, with REHIT offering additional benefits in managing hypoglycemic deviations.

### 4.5 Impact of LMICT, MIIT, and REHIT on blood lipid profiles and functional test variations in stroke patients with diabetes

Patients who have experienced a stroke should routinely undergo lipid screenings, including TC, LDL-c, HDL-c, and TG measurements (49). Current research demonstrates that stroke patients engaged in secondary preventive medication can significantly reduce their TG levels after six weeks of regular exercise, which includes activities such as assisted walking and non-weight-bearing treadmill sessions (50). This study further revealed that after four weeks of LMICT, MIIT, and REHIT training, only the REHIT group exhibited a significant decrease in TG levels compared to the CON (least squares MD = -0.43 mmol/L, 95% CI: -0.85 to -0.01, p = 0.043). This finding is consistent with the results of Cuddy et al., who observed that REHIT significantly lowers TG levels and surpasses traditional 30-minute continuous aerobic exercises at 50%-65% heart rate reserve (HRR) (51). Although the clinical benefits of reduced TG levels in this trial remain unconfirmed, a meta-analysis of 64 studies suggests a strong association between elevated TG levels and an increased risk of stroke (adjusted relative risk [RR] 1.05; 95% CI: 1.03 to 1.07), with every 10 mg/dL increase in TG corresponding to an increase in relative risk (52). TG levels are inversely related to the risk of hemorrhagic stroke (53, 54). However, similar to most exercise trials involving patients with stroke or transient ischemic attack (TIA) (16, 55–57), changes in TC, LDL-c, and HDL-c levels in the MIIT, LMICT, and REHIT groups were not significant in this study. While reducing LDL-c is recognized as a strategy to decrease recurrent stroke risk—with potential reductions of up to 20% in the five-year risk of major vascular events per 1 mmol/L—neither MIIT, REHIT, nor LMICT achieved the MD reported in D’Isabella et al.’s meta-analysis (-0.19 mmol/L, 95% CI: -0.88 to 0.50) (16, 58). This discrepancy may arise from D’Isabella’s inclusion of strength training, whereas a meta-analysis focused solely on aerobic training showed a modest post-exercise MD in LDL-c of only 0.06 mmol/L (95% CI: -0.28 to 0.15) (57), indicating the limited impact of aerobic training on LDL-c levels. Consequently, despite REHIT’s positive effect on TG levels, the overall short-term quantitative effects of LMICT, MIIT, and REHIT on lipid profiles in patients with diabetes and stroke are limited.

Furthermore, this study demonstrated a strong correlation between exercise intensity and functional recovery indices, with significant improvements in the BI and FMA. This finding aligns with prior research, which suggests that aerobic training, especially at high intensities, significantly improves functional recovery in stroke patients (59, 60). As exercise intensity escalated from LMICT to MIIT and REHIT, incremental enhancements in BI and FMA-LE were noted, with particularly significant improvements observed in the REHIT group. Surpassing a clinically significant threshold of 10 BI points indicates statistical significance and clinical relevance (61). This finding underscores the advantages of higher-intensity exercise in enhancing lower limb functional recovery compared to lower-intensity exercise, reaffirming the importance of selecting an appropriate exercise intensity for stroke patients’ rehabilitation.

## 5 Limitations of the study

Several meta-analyses published in 2020 and 2021 indicate that although numerous trials have investigated the impact of exercise on glycemic variability (GV) in patients with T2DM, systematic research on long-term training interventions (duration ≥2 weeks) remains limited (62–64). Our comprehensive literature search further corroborates this conclusion. To our knowledge, this study represents the first to employ CGM technology to continuously monitor patients with T2DM and concurrent stroke, assessing GV, MG, and TIR during a four-week randomized controlled exercise intervention while simultaneously providing data on lipid profiles and functional recovery. Although this trial demonstrated that four weeks of exercise training positively influences glycemic fluctuations, lipid profiles, and functional recovery, additional research involving more extended training and follow-up periods is crucial to fully comprehend the persistence and effectiveness of these effects. Moreover, despite the absence of statistically significant differences in medication use among the study groups, the diverse mechanisms of action of antidiabetic drugs may influence exercise-induced changes in blood glucose. Future investigations should monitor post-exercise blood glucose variability in users of various antidiabetic drugs (including insulin) to explore the interactions between these medications and exercise training and their impact on glucose management. Finally, while this study provides novel insights for patients with diabetes and stroke, the preliminary nature of these findings, attributable to the restricted sample size, underscores the necessity for more extensive studies (20). Given the small sample size of the trial, the results should be interpreted with caution. Despite these limitations, our findings offer new perspectives on personalized exercise interventions for patients with diabetes and stroke and emphasize the need for prospective clinical trials incorporating more extended intervention and follow-up periods to validate our preliminary results.

## 6 Conclusions

The findings of this study demonstrate that REHIT and MIIT significantly improve glycemic control indicators for patients with T2DM following a stroke. Data collected through CGM indicate that REHIT and MIIT can effectively reduce SD glucose, thereby reducing glucose fluctuations. It is noteworthy that REHIT has demonstrated significant advantages in reducing CV and TBR, indicating its potential benefits in correcting hypoglycemic deviations. Conversely, MIIT is more effective in lowering MG, enhancing TIR, and improving TAR for high blood glucose levels. However, the efficacy of LMICT in improving CGM-measured blood glucose control indicators is relatively limited.

Furthermore, while exercise induces modest short-term effects on lipid profiles, REHIT demonstrates a more substantial decrease in triglyceride levels compared to the control group. As exercise intensity escalates, notable enhancements in BI and FMA-LE are observed, particularly within the REHIT group, underscoring the beneficial influence of exercise intensity on facilitating functional recovery. Although these findings necessitate additional research for corroboration, they provide a valuable framework for tailored exercise prescriptions for individuals with T2DM following a stroke.

## Data Availability

The raw data supporting the conclusions of this article will be made available by the authors, without undue reservation.
